# EvoDevo meets ecology: the Ninth Okazaki Biology Conference on Marine Biology

**DOI:** 10.1186/2041-9139-4-18

**Published:** 2013-06-24

**Authors:** Ulrich Technau, Virginia M Weis

**Affiliations:** 1 Department of Molecular Evolution and Development, University of Vienna, Althanstrasse 14, Wien 1090, Austria; 2 Department of Zoology, Oregon State University, Corvallis, OR 97331, USA

**Keywords:** Cnidaria, Ctenophora, Dinoflagellate, Symbiodinium, Symbiosis, Bacterial Symbionts, Evolution

## Abstract

The “9th Okazaki Biology Conference: Marine Biology II” held at the National Institute for Basic Biology (NIBB) in Okazaki, Japan and at the Okinawa Institute of Science and Technology (OIST) in Okinawa, Japan (14–19 October 2012) bridged the fields of EvoDevo, symbiosis and coral reef ecology.

## Introduction

EvoDevo has come a long way from comparing expression patterns towards functional studies in several of the emerging new model systems. However, there is increasing awareness that the evolution of development of organisms is intimately tied to their environment and, in particular, to the close interactions with other organisms, above all symbionts and parasites. The recent international 9th Okazaki Biology Conference: Marine Biology II brought together researchers from the fields of EvoDevo, symbiosis and coral reef ecology. The meeting was organized around four broad themes: (1) cnidarians and their symbionts as holobionts/metaorganisms, (2) interactions of organisms and meta-organisms with the environment, (3) genomics and gene regulation, and (4) evolution of development in cnidarians and ctenophores. It was organized by Jun Minagawa and Kiyotaka Okada (NIBB), Nori Satoh (OIST), and Thomas Bosch (University of Kiel, Germany) and sponsored by NIBB, OIST and the Japan Science and Technology Agency (JST). The meeting was highly interdisciplinary and convened a group of broadly trained scientists with expertise spanning marine ecology, photosynthetic biochemistry, evolutionary genomics, developmental biology, microbiology and host-microbe interactions.

### Animals and their symbionts as metaorganisms

Much of the meeting centered around the advances in our understanding of the relationships between animals and their associated symbiotic microbiota. Two presentations forwarded a new paradigm of animals with their microbiota as being a critical and fundamental biological unit that has co-evolved over billions of years. Thomas Bosch (CAU, Kiel, Germany) discussed how the cnidarian *Hydra*, with its complex and diverse prokaryotic microbiota, its intracellular photosynthetic green algal eukaryote and a still-to-be-characterized virome provide an excellent model for in-depth study of a metaorganism, including how these consortia are initiated and regulated. Eugene Rosenberg (Tel Aviv University, Israel) advocated for the adoption of a related term, the holobiont, to refer to host organisms and their associated microbiota, and further argued that the hologenome is a key unit of selection in evolution.

**Figure 1:**
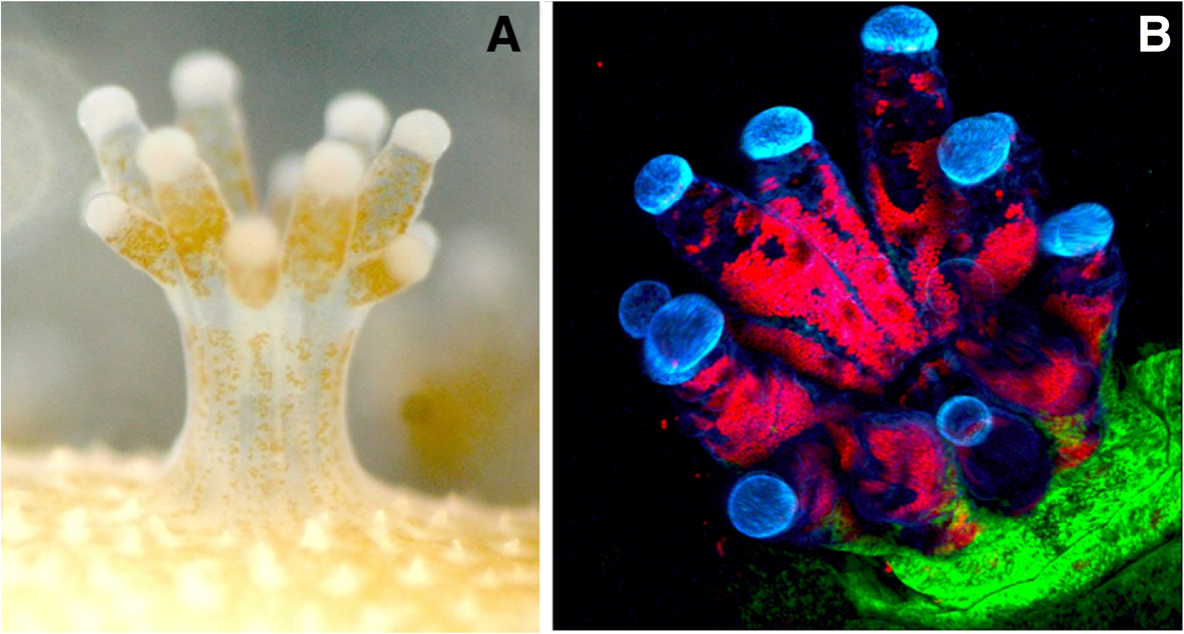
**Coral polyps in symbiosis.** (**A**) Light micrograph of an *Acropora digitifera* polyp, with an endosymbiotic *Symbiodinium* visible as multiple brown spheres in the coral tissue. (**B**) Confocal micrograph of a naturally fluorescing *Pocillopora damicornis* polyp. Red spheres are *Symbiodinium* with chlorophyll autofluoresence. Blue and green pigment is from host-derived fluorescent proteins. Photo credit: Chuya Shinzato, OIST and Christine Farrar, Hawaii Institute of Marine Biology, for *A. digitifera* and *P. damicornis* images, respectively.

Three presentations focused largely on the host side of symbiosis, with two discussing corals and cnidarian-dinoflagellate symbioses (Figure 1) and one deep sea chemoautotrophic symbioses. John Pringle (Stanford University, USA) presented progress on several fronts in the adoption of the symbiotic anemone *Aiptasia* as a model system for the study of symbiosis in corals. He described significant progress in development of genomic resources and predictable spawning of anemones in the lab. However, he also highlighted several remaining challenges, including reliable transfection, gene knockdown and development of cell culture. Virginia Weis (Oregon State University, USA) described studies on the regulation of cnidarian-dinoflagellate symbiosis. Evidence suggests that onset and maintenance of symbiosis is driven in part by host innate immune responses, including phagocytosis, the sphingosine rheostat and the TGF-beta pathway and modulation of these responses by colonizing dinoflagellates. François Lallier (Université Pierre et Marie Curie, Roscoff, France) described new work in progress on the deep-sea chemoautotrophic mussel *Bathymodiolus* that harbors both sulfide- and methane-oxidizing bacteria in its gills. His group is engaged in high throughput proteomic and transcriptomic studies aimed at describing host genes important in the symbiosis.

Three talks focused on the microbial components in metaorganisms. Tony Larkum (University of Sydney, Australia) provided a broad view of eukaryotic photosynthetic microbes and their critical role as endosymbionts in marine animals. He went on to discuss the importance of the dominant symbiont in corals, the dinoflagellate *Symbiodinium* spp., its close phylogenetic placement with apicomplexan parasites, and how it has played a critical role in coral evolution. Sebastian Fraune (University of Kiel, Germany) discussed the remarkable stability and specificity of host-microbe interactions between *Hydra* and its associated microbiota. The stability is strongly dependent on host genotype, and recent work on the role of the immunity gene MyD88 has shown that host immune response is critical in shaping the microbial community. In a presentation on chemoautotrophic bacteria-animal symbioses from deep sea hydrothermal vent ecosystems, Tadashi Maruyama (Japan Agency for Marine-Earth Science and Technology, Japan) described genomic changes in the chemoautotrophic gamma-proteobacteria in *Calyptogena* spp. clams, including changes in genome size, increasing AT bias and loss of DNA repair genes.

Masayoshi Kawaguchi (NIBB, Japan) provided an interesting departure from discussions on cnidarians with his presentation on the highly studied symbiosis between leguminous plants and nitrogen-fixing *Rhizobium*. This system is well understood and may serve as a model for animal symbioses. He described several host factors that participate in the homeostatic maintenance of the balance between host and symbiont biomass. These factors are long-distance root- and shoot-derived signals that serve to regulate root nodule (site of symbionts) number.

### Interactions of organisms and meta-organisms with the environment

Another broad area covered at the conference was environmental biology: how organisms and meta-organisms sense and respond to their environment and to environmental stress, especially related to climate change. Fourteen presenters discussed a broad array of studies in many different systems. Three investigators spoke on circadian rhythms. The timing of coral mass spawning was described by Peter Vize (University of Calgary, Canada) as including responses entrained to sunlight and moonlight as well as endogenous circadian rhythms. Using transcriptomic approaches, spawning corals were shown to exhibit entrained circadian patterns of gene expression. Similarly, Oren Levy (Bar Ilan University, Israel) discussed rhythmic expression patterns of numerous genes in the coral holobiont that are central to coral biology, including calcification and photosynthesis. This raises the important question of which partner(s) in the holobiont is controlling the rhythm. Takao Kondo (Nagoya University, Japan) provided a detailed understanding of the master pacemaker protein, KaiC, whose phosphorylation state drives circadian oscillations in cyanobacteria. Induction of settlement and metamorphosis in *Acropora* spp. planula larvae was described by Masayuki Hatta (Ochanomizu University, Japan) as being due to detection of induction cues from biofilms composed of a diverse set of bacteria on reef substrata. In addition, he detailed evidence of sensory neurons concentrated at the aboral pole that are responsible for release of neurohormones that drive metamorphosis.

Several researchers discussed the effects of light and temperature on the biology of corals and their photosynthetic dinoflagellate symbionts *Symbiodinium* spp. Peter Ralph (University of Sydney, Australia) discussed new approaches to quantifying and characterizing photoprotective mechanisms in the light harvesting pigment apparatus in *Symbiodinium* spp. Photoprotection plays a key role in the ability of symbionts to manage high light and elevated temperature stress and furthermore, it appears to vary between different *Symbiodinium* types. Similarly, in another presentation on *Symbiodinium* photosynthesis, Shunichi Takahashi (Australian National University, Australia) described differential susceptibility to photoinhibition and photobleaching from thermal stress in differing strains of *Symbiodinium*. He described his current work aimed at uncovering the mechanisms underlying this differential susceptibility and related mechanisms of thermal acclimation in *Symbiodinium* that protect against stress and reduce bleaching in the host. Work presented by Jun Minawaga (NIBB, Japan) on the chlorophyte *Chlamydomonas reinhardtii*, a classic model system for photosynthesis, is revealing a detailed understanding of the dynamic nature of molecular remodeling of the photosynthetic apparatus in response to changes in light. His group has begun to apply this knowledge and expertise to *Symbiodinium,* where they are beginning to study photosynthetic supercomplexes and how they change under stress.

Turning to a set of more ecology-based presentations, Ruth Gates (University of Hawaii, USA) described new work on patterns of *Symbiodinium* diversity among coral hosts. Her work suggests that environmental thresholds in corals are linked to the taxonomic makeup of their *Symbiodinium*, with highly specific host-symbiont partnerships, such as those in *Porites* spp., faring better during environmental stress than flexible partnerships, such as those in *Acropora* spp. Michio Hidaka (University of the Ryukyus, Japan) described similar results on coral larvae from laboratory studies showing better survival of larvae with vertically transmitted (highly specific) compared to horizontally transmitted (more flexible) symbionts. He also showed evidence that *Symbiodinium* are a burden on host larvae after a temperature stress. This may explain the high proportion of broadcast spawning species that lack symbionts in the eggs. The dynamics of larval dispersal and settlement of corals in Okinawa after the devastating mass bleaching and mortality event in 1998 was described by Kazuhiko Sakai (University of the Ryukyus, Japan). He reported that since 1998, the reefs have started to rebound due to both local recruitment (larvae traveling 10s of km) and occasional recruitment from the more distant Kerama Islands that sustained minimal bleaching damage (larvae traveling approximately 100 km).

Ann Tarrant (Woods Hole Oceanographic Institute, MA, USA) gave a presentation on the effects of anthropogenic pollutants, specifically polycyclic aromatic hydrocarbons (PAHs) in combination with high UV radiation, on cnidarian health. The anemone *Nematostella vectensis*, that commonly occurs in habitats exposed to PAHs and high UV, exhibited transcriptomic patterns of a classic cellular stress response, including up-regulation of SODs, Hsp70 and cytochrome P450s. The most dramatic responses were associated with the high UV exposure, suggesting phototoxicity.

Three participants described new work on the potentially serious threat posed by ocean acidification (OA) on coral health. OA is hypothesized to compromise the ability of corals to deposit their massive, reef-forming calcium carbonate skeletons, because carbonate chemistry is heavily dependent on pH. Denis Allemand (Centre Scientifique de Monaco, Monaco) described physiological studies on coral that show that the animal can continue to deposit CaCO_3_ in the presence of high CO_2_ by tight regulation of pH in both coral cells and within the subcalicoblastic medium where calcification takes place. This new approach gives insight into the origin of sensitivity/tolerance of corals to ocean acidification. David Miller (James Cook University, Australia) described an extensive new RNAseq study in which *Acropora millepora* was exposed to high CO_2_. Expression levels changed in 20% of host genes after short-term exposure of corals to elevated CO_2_. Many membrane-associated or secreted carbonic anhydrases showed decreased expression under elevated CO_2_, but the most dramatic impacts were on expression of genes encoding proteins of the organic matrix, many of which are coral or cnidarian-specific (that is, taxonomically restricted). In a study examining the ecophysiological impacts of OA on corals, Haruko Kurihara (University of the Ryukyus, Japan) found varying responses to increased CO_2_ levels in corals collected from different locations on reefs around Okinawa. Overall, however, she found decreased calcification rates with increased CO_2_ incubation.

### Genomics and gene regulation of cnidarians

Great advances in our understanding of symbiosis and coral bleaching are expected to come from genomic sequences of the coral host and its dinoflagellate symbiont *Symbiodinium*. A third major topic of the meeting was devoted to genomics and gene regulation of cnidarians. Recently, a team led by Nori Satoh has sequenced the genome of the scleractinian coral *Acropora digitifera*. Nori Satoh (OIST, Japan) showed interesting data from the *A. digitifera* genome that among the fast-evolving genes, genes involved in immunity and apoptosis are overrepresented. One interesting example is the NOD-domain protein, involved in inflammatory reactions and regulation of apoptosis, (for example, the presence of 500 NOD domain genes compared to about 46 in the sea anemone *Nematostella*). Chuya Shinzato (OIST, Japan) emphasized the comparison with the closely related coral *Acropora millepora*, one of the most abundant corals on the Great Barrier Reef in Australia. While the *A. digitifera* genome corroborates the view of a conserved complex gene repertoire shared with bilaterians, a substantial portion of the *Acropora* genes (about 11%) are shared only with corals. An important component in understanding the interactions between corals and their dinoflagellate symbiont *Symbiodinium*, is the sequencing of the symbiont genome. The Satoh team is currently sequencing *Symbiodinium* clade B1 originally isolated from *Montastrea faveolata*. Eiichii Shoguchi (OIST, Japan) from the Satoh team reported the current status of the sequence analysis of the 1.5Gbp large genome of this dinoflagellate. One of the remarkable features that may have contributed to the relatively large genome size of this organism is the unusually high number of introns in the nuclear genes, most of which are unidirectionally oriented in the genome. As in other dinoflagellates, the plastid genome is highly fragmented. The fragments form a number of minicircles and show extensive post-transcriptional editing. The genome sequence of a coral and its dinoflagellate symbiont will provide us with many candidate genes involved in the specific interactions and the responses to environmental stresses and immune challenges.

Another step in the understanding of how corals regulate their development, interact with symbionts and with the environment comes from the analysis of epigenetic regulation. Sylvain Forêt (Australian National University, Australia) presented the DNA methylation patterns of two distantly related cnidarians, *Acropora millepora* and *Hydra magnipapillata*. In both species, DNA methylation is concentrated in gene bodies and associated with ubiquitous gene expression, a common pattern in invertebrates and distinct from vertebrates, where methylation is mostly found in upstream silenced promoters.

### EvoDevo of cnidarians

**Figure 2:**
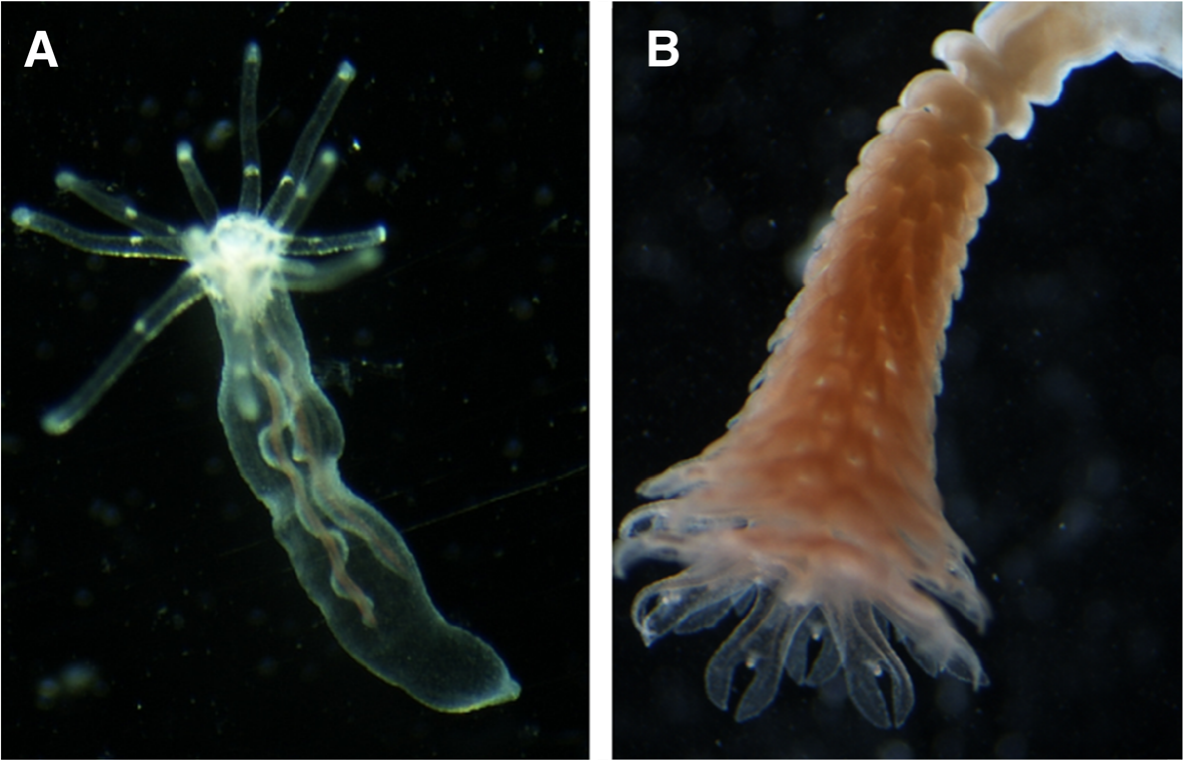
**Cnidarian model organisms useful in the study of EvoDevo.** (**A**) A juvenile anthozoan *Nematostella vectensis*. (**B**) A strobilating schyphistoma of the scyphozoan *Aurelia aurita*. Developing ephyrae are stacked, with the largest and most mature ephyra at the distal end. Photo credit: Ulrich Technau, U. of Vienna.

How development in cnidarians is regulated, which processes are conserved and which have diverged or evolved independently was the focus of the EvoDevo section of the meeting (two EvoDevo model organisms *Nematostella vectensis* and *Aurelia aurita* shown in Figure 2). A frequent misconception in the EvoDevo field arises from the misuse of the term "basal metazoan", as convincingly explained by Michael Manuel (Université Pierre et Marie Curie, Paris, France). Manuel argued that ctenophores, whose position within the phylogenetic tree is still hotly debated, show both unexpected patterns of conservation (for example, the role of Wnt signaling in stem cells and body plan of adult *Pleurobrachia*) as well as highly diverged and sophisticated anatomy and gene expression. Thus, ctenophores and other representatives of "basally branching" lineages should not be considered globally more ancestral than others, for instance, insects or vertebrates. A remarkably conserved role for Wnt signaling in early development, in particular in the formation of the primary body axis, involving a positive feedback loop of Wnt signaling, has been reported for the hydrozoan *Hydra* by Thomas Holstein (University of Heidelberg, Germany). In addition, the Holstein group could identify a putative diverged homolog of *nodal*. Functional work showed that this *Nodal-related* gene is crucial for the early steps of budding, providing the symmetry break necessary for creating a new axis perpendicular to the main body axis of the polyp. The role of canonical and non-canonical planar cell polarity (PCP) Wnt signaling was also the main topic of the presentation by Evelyn Houliston (Observatorie Océanologique, Villefranche-sur-mer, France), whose group has demonstrated that coordinated activation of these two pathways is crucial to establish polarity along the oral-aboral axis during embryonic development in the hydrozoan *Clytia hemisphaerica*. As in vertebrates, *Nematostella* and *Hydra*, *Brachyury* in *Clytia* is a conserved downstream target of canonical Wnt signaling; however, unlike in other species (see below), no feedback loop between these genes could be detected. In *Clytia* maternally localized “determinant” mRNAs not only direct Wnt pathway activation but may also have a conserved role in germ line/stem cell specification.

How gene regulatory networks act in cnidarian development might be pivotal for our understanding of how body plans evolved. Joel Smith (Marine Biological Laboratory at Woods Hole, USA) is developing high throughput transcriptome data of the first 24 hours of development of the sea anemone *Nematostella vectensis*, with high temporal resolution and also is trying to establish cell-type specific transcriptomes through a BAC-based ribosome capture procedure. Ulrich Technau (University of Vienna, Austria) reported on the target genes of a number of genes with a conserved role in mesoderm formation and differentiation in bilaterians, (for example, *Brachyury, twist, mef2*). Technau showed that *Brachyury* is involved in a conserved feedback loop with Wnt3/beta-Catenin at the blastopore lip. He also presented a comprehensive investigation of the evolutionary origin of all known bilaterian muscle proteins and their analysis in two sponges and two cnidarians, strongly suggesting that cnidarian and bilaterian striated muscles evolved independently on the basis of a core set of ancestral proteins predating the emergence of metazoans.

Metamorphosis and settlement of larvae are crucial and discernable steps in the formation of coral reefs. Eldon Ball (Australian National University, Australia) reported the dynamic expression of two neurotransmitters, RFamide and LWamide, in two non-overlapping populations of neurons. Both peptides disappear during metamorphosis, indicating a possible turnover of the nervous system. LWamide may be particularly interesting as it has been shown to drive metamorphosis in other cnidarians. The nervous system of hydrozoans was also the focus of interest in two other talks. While the nervous system of cnidarians is commonly described as diffuse and lacking symmetry, *Hydra oligactis* shows a nerve ring between mouth and tentacles. Shun Hamada (University of Fukuoka, Japan) screened this tissue by subtractive hybridization and found synapsin, a synaptic vesicle-associated protein. Synapsin colocalizes to RFamide- but not LWamide-expressing neurons in the hypostome and tentacle region, which may reflect a specific role for these neurons. Of all the known bilaterian neurotransmitters, acetylcholine (Ach) is the best studied system. Ach, PAM-like peptides, as well as numerous Ach receptor subunits are present in cnidarians; however some important members are missing (vAChT) or highly divergent (AchE). The question of whether this transmitter system has a function in cnidarians was addressed by Toshitaka Fujisawa (University of Okazaki, Japan), who investigated the expression of the existing members of the pathway in *Hydra*. Notably, although the *Hydra* AchE worked in heterologous patch-clamp systems, none of the members of the cholinergic system appears to be expressed in neurons. Therefore, it seems that the cholinergic neurotransmitter system originated in the bilaterian ancestor.

Metagenesis, that is, the transformation from a polyp into a medusoid form, occurs in species of the medusozoans (that is, Scyphozoa, Hydrozoa and Cubozoa). In the scyphozoan *Aurelia aurita*, up to 20 young medusae (ephyrae) are generated from a single polyp by a process called strobilation (Figure 2B). Konstantin Khalturin (University of Kiel, Germany) has now uncovered some of the major regulators of this process. Classical transplantation experiments showed that strobilation is inducible by a diffusible substance, identified as retinoic acid (RA), in addition to a taxonomically restricted peptide. Khalturin proposed that the role of RA in strobilation might reflect an ancestral role in metagenesis, since RA also plays a role in metamorphosis in some bilaterians (for example, tadpole-frog).

Taken together, this exciting meeting showed that previously separated fields, that is, ecology and conservation biology, evolutionary and developmental biology, genetics and cell biology are now emerging as a new synthesis, potentially under the umbrella name of EcoEvoDevo, promoted by Scott Gilbert and David Epel in a recent textbook.

The meeting has shown that new attempts are underway to tackle the intimate relationships between organisms, their symbionts and the environment. For many, if not all organisms, symbiosis often not only means a close association of two species but in fact the concept of a holobiont, that is, the close association of many different organisms from several taxonomic levels. These associations are dynamic and crucial for the shape, physiology and life cycle of an animal. They usually build up during early development, dependent on the environment. This shows the need for an integrated effort from developmental evolutionary biology and ecology in order to understand symbiosis and the holobionts. Only recently, through the advent of inexpensive high-throughput sequencing technologies, many of these problems are becoming approachable. Due to the rising data sets from these studies, research communities will require bioinformatic resource centers and servers. It is obvious that cnidarian researchers have stepped forward in this field, but researchers from other fields will surely also follow along these paths.

## Abbreviations

Ach: Acetylcholine; AChE: Acetylcholine esterase; BAC: Bacterial artificial chromosome; CAU: Christian-Albrechts-University Kiel; JST: Japan Science and Technology Agency; NIBB: National Institute for Basic Biology; NOD: Nucleotide binding oligomerization domain; OA: Ocean acidification; OIST: Okinawa Institute of Science and Technology; PAHs: Polycyclic aromatic hydrocarbons; PAM: Peptidyl-glycine alpha-amidating monooxygenase; PCP: Planar cell polarity; RA: Retinoic acid; TGF: Transforming growth factor; vAChT: Vesicular acetylcholine transporter.

## Competing interests

The authors declare that they have no competing interests.

## Authors’ contribution

UT and VMW together drafted the manuscript. All mentioned participants of the meeting have approved the manuscript before its publication. Both authors read and approved the final manuscript.

